# Studying the Nucleated Mammalian Cell Membrane by Single Molecule Approaches

**DOI:** 10.1371/journal.pone.0091595

**Published:** 2014-05-07

**Authors:** Weidong Zhao, Yongmei Tian, Mingjun Cai, Feng Wang, Jiazhen Wu, Jing Gao, Shuheng Liu, Junguang Jiang, Shibo Jiang, Hongda Wang

**Affiliations:** 1 State Key Laboratory of Electroanalytical Chemistry, Changchun Institute of Applied Chemistry, Chinese Academy of Sciences, Changchun, Jilin, China; 2 University of Chinese Academy of Sciences, Beijing, China; 3 Key Laboratory of Medical Molecular Virology of Ministries of Education and Health, Shanghai Medical College, Fudan University, Shanghai, China; 4 Lindsley F. Kimball Research Institute, New York Blood Center, New York, New York, United States of America; University of the Witwatersrand, South Africa

## Abstract

The cell membrane plays a key role in compartmentalization, nutrient transportation and signal transduction, while the pattern of protein distribution at both cytoplasmic and ectoplasmic sides of the cell membrane remains elusive. Using a combination of single-molecule techniques, including atomic force microscopy (AFM), single molecule force spectroscopy (SMFS) and stochastic optical reconstruction microscopy (STORM), to study the structure of nucleated cell membranes, we found that (1) proteins at the ectoplasmic side of the cell membrane form a dense protein layer (4 nm) on top of a lipid bilayer; (2) proteins aggregate to form islands evenly dispersed at the cytoplasmic side of the cell membrane with a height of about 10–12 nm; (3) cholesterol-enriched domains exist within the cell membrane; (4) carbohydrates stay in microdomains at the ectoplasmic side; and (5) exposed amino groups are asymmetrically distributed on both sides. Based on these observations, we proposed a Protein Layer-Lipid-Protein Island (PLLPI) model, to provide a better understanding of cell membrane structure, membrane trafficking and viral fusion mechanisms.

## Introduction

The cell membrane, also termed the plasma membrane, plays a crucial role in various cellular activities, such as signal transduction, membrane trafficking, as well as energy conversion [1]–[4]. Although different cell membrane models have been introduced over the past century, we are still far from fully understanding this important cellular component [5]–[7].

The structure of the cell membrane was initially viewed as a sandwich that consists of protein-lipid-protein [8]. Then, based on investigations with ultrathin section electron microscopy, an improved unit membrane model was developed, indicating the presence of a lipid bilayer with a thickness of 3.5 nm, in addition to proteins [9]. With the realization of dynamic protein distribution in the cell membrane, the fluid mosaic model was introduced and has become the most accepted model until now. The fluid mosaic model highlights the aspects of “diffusion” and “mosaicism”, emphasizing that 1) both lipids and proteins are dynamic and diffuse randomly in the homogeneous lipid bilayer and 2) proteins are asymmetrically distributed in the cell membrane [5].

New evidence, however, shows that the distribution of proteins is not random and that lateral diffusion is restricted by the interaction of the membrane-bound receptors with cytoskeleton or cytosolic molecules, indicating a lateral heterogeneity in the membranes [10]. The presence of protein clusters at different scales has also been revealed in cell membranes [10]. Both proteins and lipids are important in maintaining the structure of cell membranes, but proteins occupy a larger area than expected. Thus, cell membrane structure should be considered “mosaic”, i.e., an assemblage of small pieces, and not “fluid”, as emphasized in the dynamically structured mosaic model [11]. In addition, since membrane patches and thickness are variable, it is proposed that the cell membrane is at the transition between the lipid-ordered phase and the lipid-disordered phase [12].

Based on the studies of apical membrane trafficking, virus entry into cells, and detergent-resistant membranes in both model and plasma membranes [13], the concept of lipid rafts has been introduced [6]. Lipid rafts are hypothesized to be dynamic and functional nanoscale domains that are enriched with sphingolipid, cholesterol and proteins [14]. The lipid raft model emphasizes lipids as the solvent of proteins, but also their involvement in the lateral heterogeneity of the cell membrane. Since the sizes of lipid rafts are beyond the resolution of light microscopy, studying the nature of lipid rafts is a challenging topic. Recently, along with the development of single molecule techniques, lipid rafts have been proved to work as a functional domain in the red blood cell membrane [15].

Although previous models have successfully interpreted some functions of the cell membrane, no consensus has been reached that conclusively explains the nature of the cell membrane structure by the lack of direct and *in situ* evidence. Meanwhile, these models mainly focus on single proteins and isolated protein domains but not the whole cell membrane structure in a way that would accurately describe the total protein distribution in both leaflets of the cell membrane and the interactions among membrane proteins. Traditionally, scanning electron microscopy (SEM), nuclear magnetic resonance (NMR), applying immunogold staining (IGS) to transmission electron microscopy (TEM), electron spin resonance and fluorescence microscopy have been used to study the cell membrane [9], [16]–[18]. However, the direct investigation of the structure of nucleated mammalian cell membranes under native conditions at molecular resolution has not been achieved by these techniques.

Atomic force microscopy (AFM) has become an important tool in bionanotechnology [19]. It can image biological samples under aqueous solutions with nanometer resolution without damaging the samples. The topography and structure of proteins, nucleic acid, cellular membranes and cells have been investigated at the single-molecule level with AFM [20], [21]. In addition, single-molecule force spectroscopy (SMFS) based on AFM is a highly sensitive method to measure the inter- or intramolecular forces down to piconewton level [22], [23]. It has been successfully employed to investigate the specific interactions and binding kinetics between antibody-antigen, receptor-ligand, avidin-biotin and other biological systems [24], [25].

Single-molecule fluorescence microscopies have contributed to the study of the cell membrane structure. Total internal reflection fluorescence microscopy (TIRFM) has significantly improved the signal-to-noise in single-molecule fluorescence imaging, and it has been successfully applied to image cell membrane components and dynamic events occurring at the cell surface [26], [27]. The recently developed super resolution fluorescence microscopy, stochastic optical reconstruction microscopy (STORM), has broken the diffraction barrier of light. It can resolve the fine structures and dynamic processes that cannot be achieved with conventional fluorescence microscopy [28]. STORM has achieved super resolution in three dimensions, including 20 nm in XY and 50 nm in Z, with multicolor colocalization [29]. The super resolution images of microtubules, mitochondria and clathrin-coated pits have been acquired, demonstrating that STORM is a powerful tool for cell imaging [30].

In this work, we utilized AFM, STORM and SMFS to carry out an *in situ* study of the membrane structure of nucleated mammalian cells at the single-molecule level without any fixation or severe treatment. As a result, the asymmetry of the protein distribution pattern was revealed, allowing us to propose a novel Protein Layer–Lipid–Protein Island (PLLPI) model of the cell membrane.

## Results

### The smooth ectoplasmic side of nucleated mammalian cell membranes revealed by *in situ* AFM

**Figure 1:**
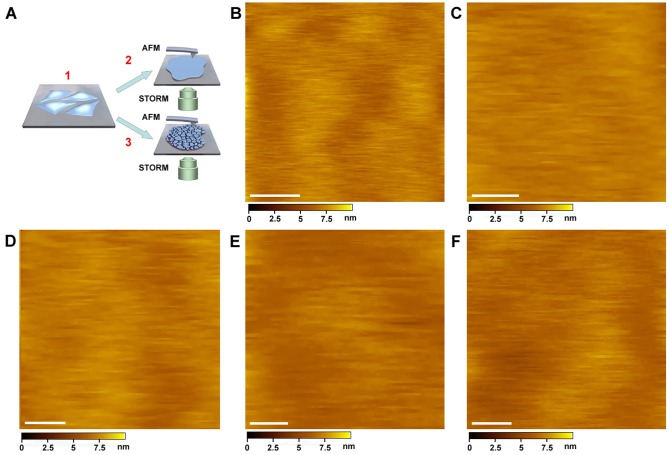
Imaging the ectoplasmic side of the cell membranes from various types of mammalian cells. (A) The scheme of this work. Cells were cultured on cover slips (A1), (A2) and (A3). The ectoplasmic and cytoplasmic sides of membranes were prepared separately and then investigated with AFM imaging, single-molecule force spectroscopy (SMFS), and STORM, respectively. (B) The ectoplasmic side of MDCK cell membrane was directly imaged on a living cell. (C) The image of the ectoplasmic side of the cell membrane (MDCK cells) prepared by shearing open the cells on a cover slip. (D) The image of the ectoplasmic side of the cell membrane (MDCK cells) prepared by centrifugation. (E and F) The ectoplasmic side of A549 (E) and HeLa (F) cell membranes prepared by the shearing open approach, respectively. Scale bars: 100 nm in (B–F).

To study the cell membrane structure, the ectoplasmic and cytoplasmic side of membranes were prepared as described previously [31] and subjected to AFM, SMFS and STORM imaging (Figure 1A), respectively.

We first studied the ectoplasmic surface of MDCK cells. In order to verify the feature of the native cell membrane, we directly imaged the ectoplasmic surface of living cells. Surprisingly, the ectoplasmic surface of MDCK cells seemed to be very smooth without obvious protrusion (Figure 1B). The roughness of membrane surface was only 1.1±0.2 nm. Cultured cells were soft and elevated by several micrometers, making it difficult to achieve high resolution; therefore, we took advantage of two other strategies, specifically shearing open and centrifuging, to obtain quasi-native cell membranes. Figure 1C showed the high resolution image of the cell membrane's ectoplasmic side prepared by shearing open the cells. The ectoplasmic surface was found to be rather smooth with a roughness root mean square (RMS) of 0.9±0.2 nm. No indents or particles were visible. As shown in Figure 1D, the ectoplasmic side of the cell membrane obtained by the hypotonic-centrifugation procedure presented a roughness RMS of 1.0±0.2 nm, thus showing a feature very similar to that in Figure 1B and 1C and providing additional evidence of the smooth ectoplasmic surface of MDCK cell membranes. To know whether this phenomenon was common for other types of nucleated mammalian cells, we imaged the ectoplasmic side of two other human cancer cell lines derived from different organs, A549 cells from lung and HeLa cells from cervix, by the shearing open approach. As shown in Figure 1E and 1F, the ectoplasmic sides of these cell membranes were as smooth as those of MDCK cells. To test our technique, we previously decorated antibodies on the cell membranes, and the result showed that the resolution of AFM was high enough to distinguish protruding proteins from the surface of cell membranes [32]. Meanwhile, it should be noted that we imaged the native or quasi-native cell membranes (unfixed), which allowed us to detect the original state of cell membranes. We have found that the fixation of the cell membrane (e.g. fixed by aldehydes) would destroy the membrane structure and induce protein aggregation. Taken together, these results demonstrate that the ectoplasmic side of native nucleated mammalian cell membranes is smooth without any obvious protrusion.

### Existence of a dense protein layer covering a lipid bilayer

**Figure 2:**
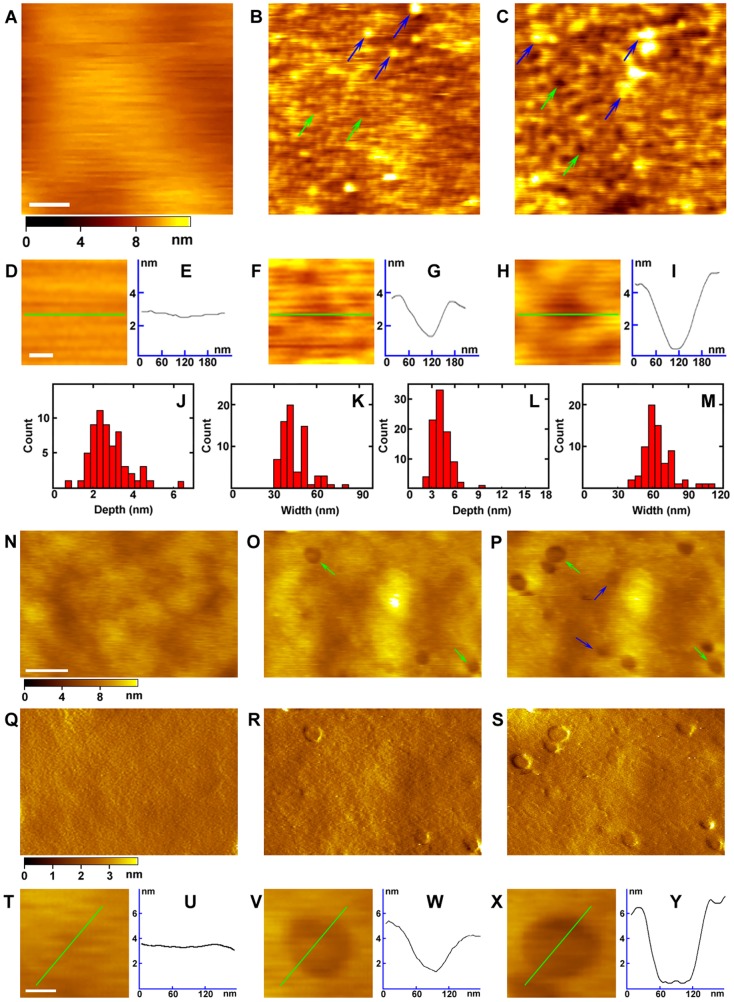
Digestion of the ectoplasmic side of the cell membrane with proteinase K or collagenase 3. (A) The AFM topographic image of the ectoplasmic side of the cell membrane. (B and C) The AFM topographic image of the ectoplasmic side of cell membranes treated with proteinase K (B) and MβCD (C) in sequence. (D, F and H) The magnified images from (A, B and C), respectively, showing the gradual deepening of the pits. (E, G and I) The cross section analysis along the green lines in (D, F and H), respectively. (J and K) The depth and width distributions of the pits after proteinase K treatment, respectively. (L and M) The depth and width distributions of the pits after MβCD treatment, respectively. (N) The AFM topographic image of the ectoplasmic side of the cell membrane without treatment. (O and P) The AFM topographic image of the ectoplasmic side of the cell membrane treated *in situ* with collagenase 3 and MβCD in sequence, respectively. (Q, R and S) The AFM amplitude images corresponding to (N, O and P), respectively. (T, V and X) The magnified images of (N, O and P), respectively. (U, W and Y) The cross section analysis along the green lines in (T, V and X), respectively. Scale bars: 300 nm in (A–C), 80 nm in (D, F and H), 300 nm in (N–S), and 50 nm in (T, V and X).

It is well established that proteins, such as receptors and glycosyl phosphatidyl inositol-anchored proteins (GPI-APs), are present on the ectoplasmic surface of the cell membrane. To verify the location and organization of these membrane proteins, we treated the ectoplasmic side of the cell membrane with proteinase K, which can digest most proteins above the lipid bilayers, and monitored the real-time changes with time-lapse AFM. The surface of undigested ectoplasmic side of the cell membrane was consistently smooth without any pits or protrusions, as indicated in the magnified image and the corresponding section analysis (Figure 2A, 2D and 2E). After digestion with proteinase K, most proteins were removed, except some undigested or half-digested proteins, as indicated by the blue arrows in Figure 2B and 2C, at the ectoplasmic side of the cell membrane. The heights between the undigested proteins and the local pits, as indicated by the green arrows in Figure 2B, ranged from 1.3 nm to 5 nm, with the majority around 2.8±0.9 nm (n = 60, Figure 2B and 2J and Table 1). The width of the pits varied from 30 nm to 80 nm, with the majority around 43.8±7.7 nm (n = 70, Figure 2B and 2K).

**Table 1:** Membrane height after treatment with enzyme and solvent.

	EP		CP	
	proteinase K	collagenase 3	proteinase K	trypsin
D_enzyme_	2.8	4		
D_MβCD_/D_TX_	4.2	4	4.3 or 8.3	
H_enzyme_			8.0	8.0
H_MβCD_/H_TX_			8.0	4.7

EP, ectoplasmic side of membrane; CP, cytoplasmic side of membrane; D_enzyme_, depth of the pits after enzyme digestion; D_MβCD_/D_TX_, depth of the pits after treatment with MβCD or TX (Triton X-100); H_enzyme_, height of the membrane after digestion by enzyme; H_MβCD_/H_TX_, height of the membrane after treatment with MβCD or TX; Unit: nm.

In order to know where the lipid bilayer is located, the digested membrane was further treated with methyl-beta-cyclodextrin (MβCD) that can deplete cholesterol in the lipid bilayer [33]. After MβCD treatment, the total height of the membrane remained the same as the untreated membrane, but the depth and width of the indents had increased, as can be observed in the magnified image and the section analysis (Figure 2F, 2G, 2H and 2I). The depths of the indents eroded by MβCD range from 2 nm to 10 nm, with the majority about 4.2±1.1 nm (n = 90, Figure 2L and Table 1), which agrees with the height of the lipid bilayer [34]. The indent widths exhibit variability between 40 nm and 110 nm, with the majority of indentations having an average width of around 63.1±10.1 nm (n = 70, Figure 2M), which was larger than that digested by proteinase K. These results demonstrate that the ectoplasmic side of the cell membrane comprises a layer of dense proteins, e.g., GPI-APs and the extracellular segment of the transmembrane proteins, associated with the lipid bilayer.

To further verify the relationship between the dense protein layer and the lipid bilayer, the ectoplasmic side of the cell membrane was digested with a more specific enzyme, collagenase 3, which can specifically digest certain membrane receptors at the extracellular side [35]. Figure 2N shows the untreated ectoplasmic side of the cell membrane, and the magnified image from Figure 2N is shown in Figure 2T. After treatment with collagenase 3, a few round pits appeared in the membrane, as indicated by the green arrows in Figure 2O, which can be distinguished more clearly from the corresponding amplitude image (Figure 2Q, 2R) and the section analysis (Figure 2U, 2W). The depth of pits was about 4 nm, which was consistent with the height of the extracellular segment of transmembrane proteins, e.g., G protein-coupled receptor [36]. After the membranes were further treated with MβCD to extract the cholesterol domains, these pits were deepened by about 3–4 nm (Figure 2P, 2Y and Table 1), showing a height similar to that of the lipid bilayer and corresponding well with the result of proteinase K-MβCD treatment. The widths of the pits were also extended by about 20 nm (Figure 2V, 2X). Newly produced pits, as indicated by the blue arrows in Figure 2P and corresponding amplitude image (Figure 2S), were also caused by MβCD treatment. These results further reveal that the proteins at the ectoplasmic side of the cell membrane form a dense protein layer with a thickness of about 4 nm and that it sits on top of the lipid bilayer.

### The domains of carbohydrates on the ectoplasmic side of membrane detected by STORM

**Figure 3:**
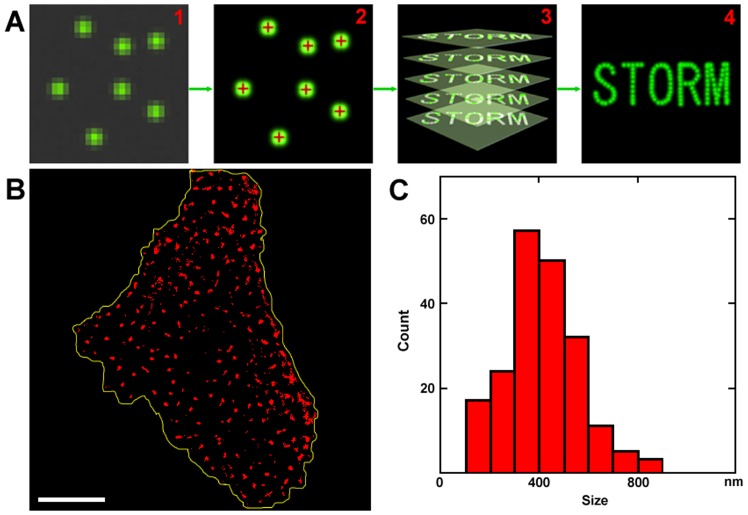
Detecting the domains of carbohydrates on the ectoplasmic surface by STORM. (A) The principle of STORM. (B) The STORM image of mannose clusters labeled with MNA-Cy5 on the ectoplasmic surface of the cell membranes. (C) The size distribution of the mannose clusters. Scale bar: 8 µm in (B).

Most of the membrane proteins, such as receptors, on the ectoplasmic surface of cells are glycosylated. These glycoproteins play important structural and functional roles in cellular activities, such as cell-cell recognition and adhesion [37]. In order to precisely localize the carbohydrates, we utilized a super resolution microscopy known as STORM. As shown in Figure 3A, the principle of STORM is based on the highly accurate positioning of photoswitchable fluorophores. In each imaging cycle, only a fraction of fluorophores was activated (Figure 3A1), making it possible to precisely localize their positions (Figure 3A2). Other fluorophores can be localized by repeating these cycles (Figure 3A3), and the overall images can be reconstructed according to the positions of these fluorophores (Figure 3A4) [38].

Mannose is one of the common carbohydrates on the membrane surface. The cell membranes were labeled with lectin MNA that can specifically recognize mannose and then imaged with STORM. As depicted in Figure 3B, mannose clusters on the ectoplasmic side of the cell membrane were plentiful and obvious. Most mannose clusters appeared to be distributed in microdomains, ranging from 105 to 850 nm, with a majority around 381±158 nm (Figure 3C). Because carbohydrates are incorporated with proteins, the distribution of carbohydrate microdomains implies that functional glycoproteins, such as receptors and transporters, may form microdomains in membranes to fulfill their functions efficiently.

We further digested the ectoplasmic side of the cell membranes by PNGase F that can cleave most saccharides from glycoproteins, and *in situ* observed the changes by AFM (Figure S3). Unlike the ectoplasmic side of human erythrocyte membranes [32], after digestion by PNGase F, the ectoplasmic side of MDCK cell membranes exhibited no apparent pits or indents on the smooth surface. This result indicates the absence of a dense layer of saccharides on the ectoplasmic surface of the cell membranes, consistent with the patchy distribution of carbohydrates on the membrane surface shown by STORM imaging.

### The rough cytoplasmic side of nucleated mammalian cell membrane imaged by *in situ* AFM

**Figure 4:**
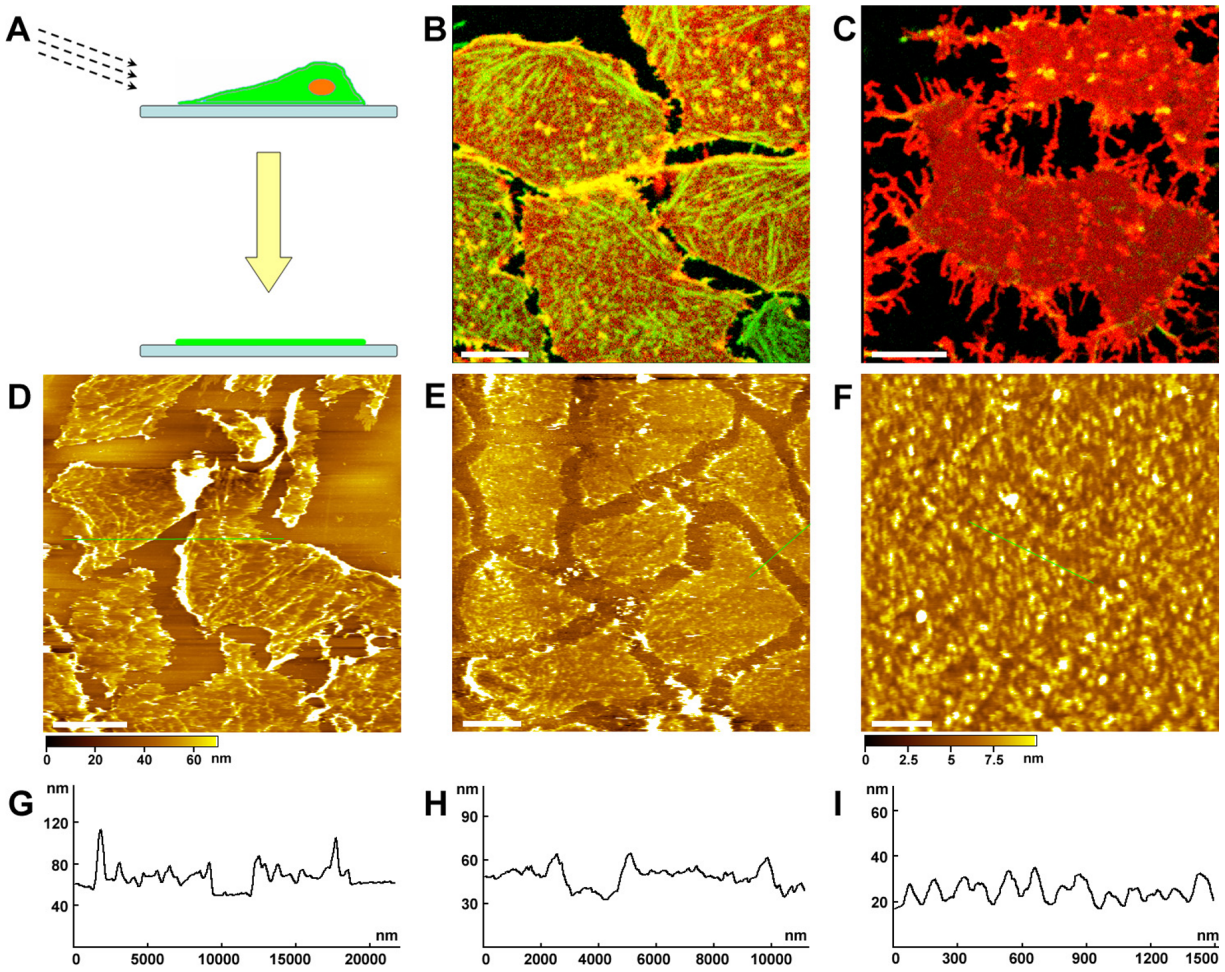
Imaging the cytoplasmic side of the cell membrane. (A) The scheme for preparing the cytoplasmic side of the cell membrane. (B and C) The fluorescent images of the cytoplasmic side of the cell membrane before and after incubation with high-salt buffer, respectively. The red membrane patches represent the lipid bilayer labeled with DiI, and the green fibers represent the actin filaments labeled with phalloidin-FITC. (D and E) The AFM topographic images of the cytoplasmic side of the cell membrane before and after incubation with high-salt buffer, respectively. (F) The high magnification image of the cytoplasmic side of the cell membrane. (G–I) Cross section analysis along the green line in (D–F), respectively. Scale bars: 7 µm in (B)–(E); 500 nm in (F).

The distribution of proteins at the cytoplasmic side of the cell membrane is another key aspect of cell membrane structure and function. Various types of proteins can be found at the cytoplasmic side of the cell membrane, such as the intracellular domains of receptors and transporters. To achieve high-resolution imaging of the cytoplasmic side of the cell membrane by AFM, the cells were sheared open by hypotonic buffer (Figure 4A), followed by hypertonic salt treatment that removed the membrane skeletons and non-transmembrane proteins. Since the transmembrane proteins were inserted in the lipid bilayer, they were not removed by hypertonic buffer, as expected [31]. Figure 4B displays the fluorescent image of the cytoplasmic side of the cell membrane, in which abundant actin filaments (green) are visible on the membrane surface (red). The cytoskeletons were disrupted by high-salt treatment (Figure 4C). The AFM topographical images of the cytoplasmic side of membranes before and after treatment with high-salt buffer are shown in Figure 4D and 4E, respectively. Dense actin filaments are shown as strips (Figure 4D and 4G), while no obvious cytoskeleton can be observed in Figure 4E. The average height of the membranes was 19.5±2.8 nm (Figure 4H). The cytoplasmic side of cell membranes was rather rough and covered with proteins, which can be seen more clearly in the magnified image (Figure 4F). The roughness RMS of the cytoplasmic side of membranes was 3.7±0.2 nm (Figure 4F), which was much more significant than that of the ectoplasmic side (Figure 1). The height of the proteins measured from top to bottom was 11.2±1.9 nm (Figure 4I). Based on the similarity of heights of the ectoplasmic protein layer and lipid bilayer both at about 4 nm (Figure 2), the total height of the cell membrane was calculated to be about 20 nm, consistent with the real size measured from the whole cell membrane (Figure 4E). The width of the protein microdomains was 98.5±8.6 nm, much larger than that of a single protein, about 20 nm measured by AFM [39], indicating the presence of multiple proteins in the microdomains. The distribution of distances of the adjacent protein domains from border to border was about 53.2±12.0 nm. These results demonstrate that the cytoplasmic side consists of protein microdomains scattered in the lipid bilayer.

### Dissecting the membrane architecture by treating the cytoplasmic side of membrane with trypsin and Triton X-100

**Figure 5:**
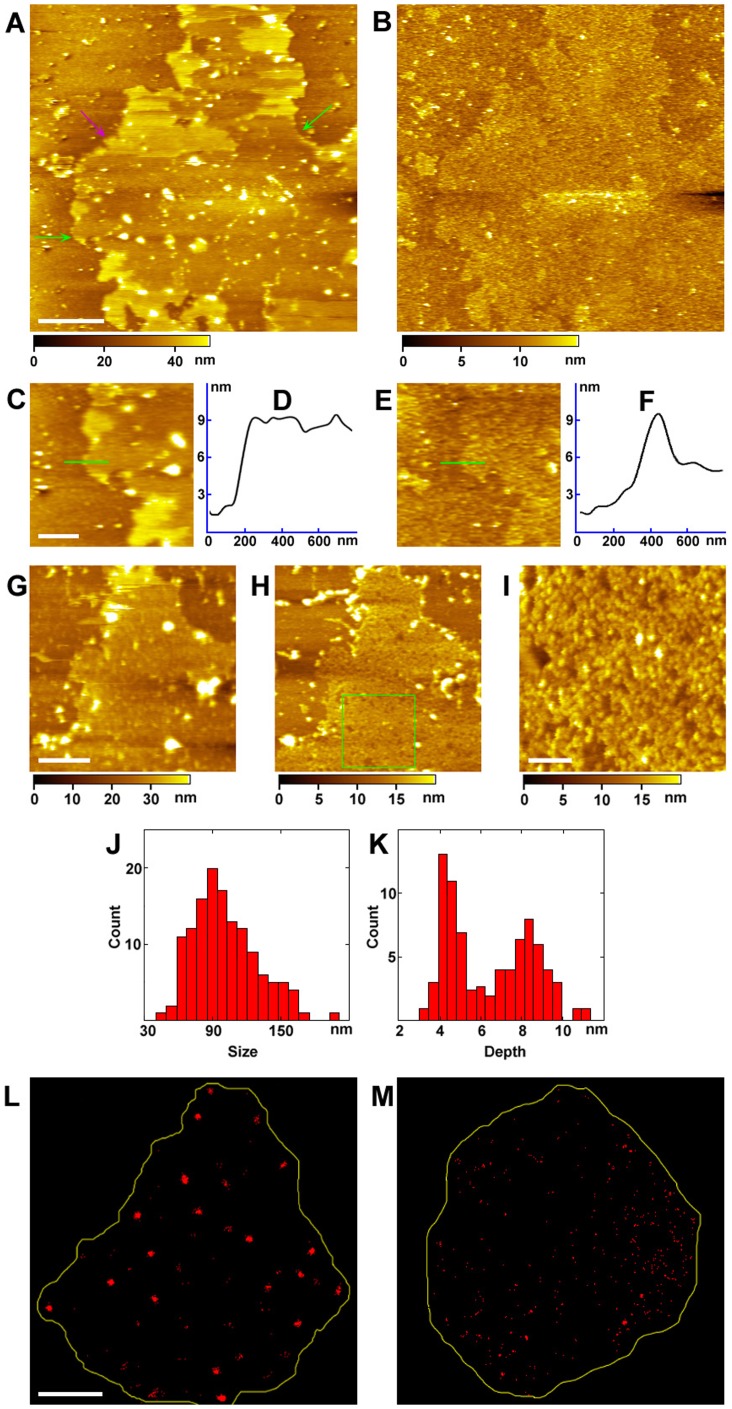
The cytoplasmic side of the cell membrane treated with trypsin or proteinase K. (A) AFM image of the cytoplasmic side of the cell membrane after digestion with trypsin for 1 h. The single and double layers of membranes are indicated by green and pink arrows, respectively. (B) The membranes were treated *in situ* with 0.1% Triton X-100. (C) and (E) The magnified images from (A) and (B), respectively. (D) and (F) The cross section analysis along the green lines in (C) and (E), respectively. (G) and (H) The images of the cytoplasmic side of membranes after treatment with proteinase K and MβCD in sequence. (I) The magnified image of the green square area in (H). (J) and (K) The size and depth distributions of the pits eroded with MβCD in (H and I), respectively. (L) and (M) The STORM images of band 3 labeled with anti-band 3-Cy5 on the cytoplasmic side of the cell membrane before and after treatment with MβCD, respectively. Scale bars: 2 µm in (A–B), 200 nm in (C–E), 1 µm in (G–H), 300 nm in (I), and 4 µm in (L–M).

To investigate the relationship between the protein microdomains and lipid bilayer, the cytoplasmic side of membranes was treated with trypsin that could digest most membrane protein domains at the cytoplasmic side. The topographical image of the digested cytoplasmic side of the cell membrane showed that most of the proteins had been removed, thereby revealing the relative smoothness of local membrane patches (Figure 5A, 5C). Some undigested proteins were right above the lipid bilayer, as shown by the bright dots. The height of single-layered, digested membrane patches was 8.0±0.5 nm (average time n = 30, Figure 5D, Table 1), as depicted by the green arrows in Figure 5A, indicating that the membrane patches are composed of the lipid bilayer (4 nm) and a dense protein layer (4 nm) at the ectoplasmic side, as mentioned previously. Double layers of digested membranes with an average height of 15.7±1.9 nm (n = 18), as indicated by the pink arrow, can still be seen.

Triton X-100 has been widely used to destroy the lipid bilayer by interacting gently with the lipids. We then used 0.1% Triton X-100 to treat the trypsin-digested cytoplasmic side of the cell membrane and disrupted the lipid bilayer (Figure 5B). As a result, the average height of the remaining membrane decreased to about 4.7±0.4 nm (Figure 5B, 5F and Table 1), which was consistent with the height of the protein layer on the ectoplasmic side of membrane. The proteins (Figure 5B and 5E) on the remaining membrane surface may consist of membrane-anchoring proteins, such as GPI proteins, while the pits in the left membrane implicated sites of the transmembrane proteins, such as receptors. These results further confirm that the whole cell membrane consists of inner dispersed protein domains (12 nm), a lipid bilayer (4 nm), and an ectoplasmic layer of dense proteins (4 nm).

### Directly verifying the existence of the cholesterol-enriched domains by treating the cytoplasmic side of the cell membrane with proteinase K and MβCD

To directly clarify whether cholesterol-enriched domains, i.e., lipid rafts, exist on the cytoplasmic side of the cell membrane, we then treated the cell membrane with proteinase K. Figure 5G shows the cytoplasmic side of the cell membrane after digestion by proteinase K. The left membrane was smooth with a height of 8.0±1.4 nm (Table 1), which agreed perfectly with the result of trypsin treatment (Figure 5A). Next, we treated *in situ* the exposed lipid layer by MβCD to eliminate the cholesterol-enriched domains. After MβCD was injected *in situ* into the AFM sample cell, the lipid bilayer was quickly eroded, as shown in Figure 5H. The height of the left membrane patches remained the same (about 8 nm, as shown in Table 1), except for many pits. The magnified image of the green square area is shown in Figure 5I. The pits eroded by MβCD were 40–200 nm in size, with the majority around 98.5±25.5 nm (Figure 5J), which was in good agreement with the sizes of the protein domains on the cytoplasmic side of the cell membrane, as shown in Figure 4F. The depth distribution of the areas eroded by MβCD is shown in Figure 5K. Two major depth distributions of the pits are evident: one is at 4.3±0.3 nm (Table 1), corresponding with the height of the lipid bilayer, and the other one is at 8.3±0.4 nm, consistent with the total height of the lipid bilayer and ectoplasmic protein layer. Taken together, our data indicate that the cholesterol-enriched domains may be the protein microdomains on the cytoplasmic side of cell membranes (Figure 4F).

### The relationship between band 3 and cholesterol-enriched domains revealed by STORM

Cholesterol-enriched domains in the cell membranes are proposed to perform various functions through embedded proteins [14]. We attempted to locate the functional proteins associated with cholesterol-enriched domains. Band 3 serves as an ion transporter and the anchoring sites for ankyrin, protein 4.1, aldolase and other membrane-bound proteins, and it has also been found to be involved in the regulation of cell shape and flexibility [40]. Although several studies have reported on the relationship between band 3 and cholesterol-enriched domains, direct observation at high resolution has not thus far been achieved [41]. Here, band 3 was localized at the cytoplasmic side of membranes using the super-resolution fluorescence microscopy afforded by STORM.

Figure 5L shows the fluorescence images of band 3 on the cytoplasmic side of the cell membrane. Most band 3 proteins tended to form microdomains with the size of 443±65 nm. After the cytoplasmic side of membranes were treated with MβCD, the amount of band 3 decreased quite obviously, and the cholesterol-enriched domains remained in a dispersed state without large domains (Figure 5M). This undoubtedly indicates the presence of cholesterol-enriched domains on the cytoplasmic side of the cell membrane and demonstrates that band 3 was localized in cholesterol-enriched domains. Other important membrane proteins, such as ATPase and EGF receptor, were also confirmed to be associated with cholesterol-enriched domains by STORM and molecule recognition imaging (Figure S1 and S2).

### The asymmetry of the exposed amino groups on both sides of membranes detected by AFM force spectroscopy

**Figure 6:**
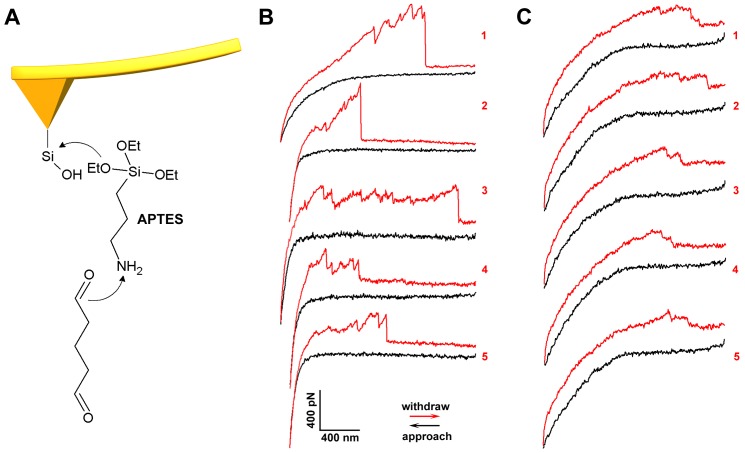
Detecting the amino groups on both sides of the cell membrane by single- molecule force spectroscopy. (A) The scheme of AFM tip functionalized with aldehyde group. (B and C) The typical force curves acquired on the cytoplasmic and ectoplasmic side of the cell membrane, respectively. The black and red lines represent the approaching and withdrawn curves, respectively.

In order to confirm the exposure of proteins on the cytoplasmic and ectoplasmic sides of cell membranes, single-molecule force spectroscopy was applied to detect the amino groups on the surface of the cell membrane. The scheme of AFM tip functionalization is shown in Figure 6A. The aldehyde group linked onto the AFM tip could bind the exposed amino groups of membrane proteins, and this interaction was recorded in AFM force curves. The typical force curves acquired at the cytoplasmic and ectoplasmic sides of cell membranes, out of thousands of force curves, were shown in Figure 6B and 6C, respectively. In Figure 6B, multiple force events were evident in these force curves, and the maximum unbinding forces could reach about 400 pN at a loading rate of 0.72 nN/s. However, only two or three force events were evident in the force curves in Figure 6C, and the maximum unbinding forces were less than 100 pN at a loading rate of 10.9 nN/s. The overall binding probabilities, i.e., the number of all recorded force curves divided by the number of force curves with the unbinding events, at the cytoplasmic and ectoplasmic sides of membranes were 94.5% and 36.9%, respectively. These results reveal that a large quantity of exposed amino groups are present on the cytoplasmic side, while fewer amino groups are present on the ectoplasmic side of membranes, essentially because most proteins on the ectoplasmic side of the cell membrane are glycosylated and compacted, while considering that there is a denser protein layer in the ectoplasmic side than that in the cytoplasmic side.

## Discussion

**Figure 7:**
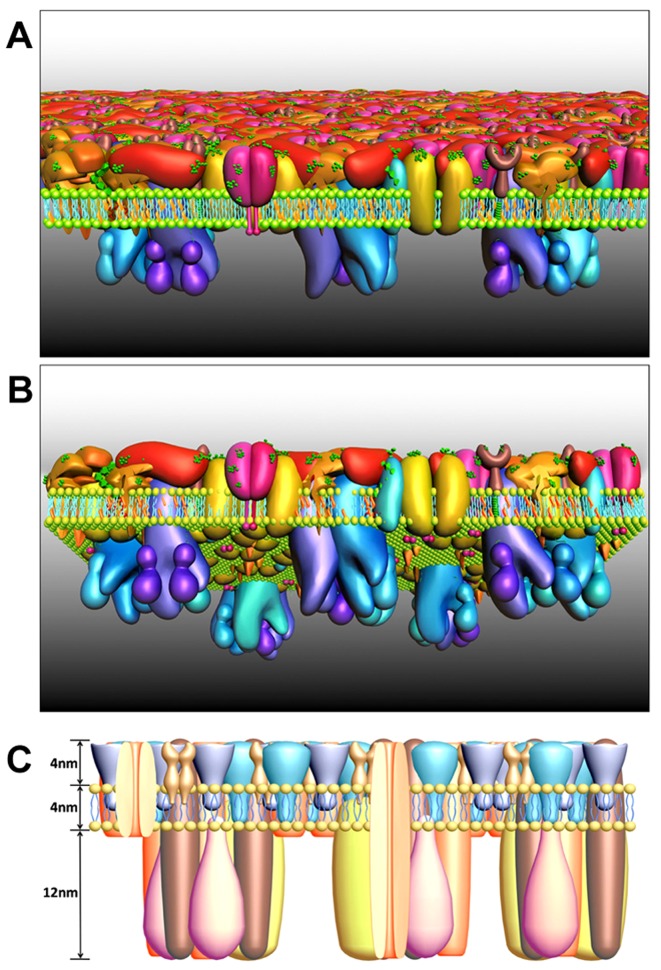
The proposed Protein Layer-Lipid-Protein Island (PLLPI) model of the cell membrane. (A and B) The top and bottom view of the cell membrane, respectively. The proteins on the ectoplasmic side of the cell membrane form a dense protein layer to show a smooth feature (A); the proteins on the cytoplasmic side tend to form dispersed microdomains (B). (C) The size of the cell membrane. The total height of the cell membrane is 20 nm, which is composed of the ectoplasmic protein layer (4 nm), lipid bilayer (4 nm) and cytoplasmic protein layer (12 nm).

Using a combination of single-molecule techniques, including AFM, SMFS and STORM to study the structure of nucleated cell membranes *in-situ*, we found that (1) proteins at the ectoplasmic side of membrane form a dense protein layer (4 nm) on top of a lipid bilayer; (2) proteins aggregate to form islands evenly dispersed at the cytoplasmic side of the cell membrane with a height of 10–12 nm; (3) cholesterol-enriched domains exist in the cell membrane; (4) carbohydrates stay in microdomains at the ectoplasmic side; and (5) exposed amino groups are asymmetrically distributed on both sides. These observations lead us to propose an improved structure model of nucleated mammalian cells, the Protein Layer–Lipid–Protein Island (PLLPI) model (Figure 7). Proteins are asymmetrically distributed on the cell membrane surface. The ectoplasmic side of the cell membrane consists of various types of proteins, such as extracellular segments of receptors and the GPI-APs, above the lipid bilayer. The proteins at the ectoplasmic side of the cell membrane form a dense protein layer showing a smooth feature (Figure 7A) with a height of about 4 nm (Figure 7C). The cytoplasmic side of the cell membrane is relatively rough, in which proteins tend to form protein domains, most likely cholesterol-enriched domains, with a height of about 12 nm (Figure 7B, 4F and 7C). Current cell membrane models, such as the liquid mosaic model, were proposed mainly based on the results of transmission electron microscopy, which involved physicochemical treatments of biological specimens, such as fixation, dehydrating, embedding, sectioning, staining, or extreme conditions, including high vacuum and low temperature, thus failing to provide the ultrastructure of the cell membranes in their native state. In contrast, the PLLPI model is proposed on the basis of observations by *in-situ* single-molecule techniques, including AFM, STORM and SMFS, which provide the least disturbance to the native membrane organization. Therefore, the PLLPI model may be much closer to the native structure of cell membranes.

This model emphasizes the asymmetry of the cell membrane. On the ectoplasmic side of membrane, the proteins exist so close that the ectoplasmic surface appears very smooth, while proteins on the cytoplasmic side aggregate to form microdomains, or cholesterol-enriched domains. This asymmetry is further verified by single-molecule force spectroscopy, which indicates that the amino groups on the cytoplasmic side of the cell membrane are exposed, while the ectoplasmic side is covered with oligosaccharides. Cell membrane asymmetry is consistent with its basic components and characteristics. Innate asymmetry can be found in the composition of lipids, proteins and cholesterol between the ectoplasmic and cytoplasmic sides of cell membranes; for instance, various types of GPI-APs are present on the ectoplasmic side of the cell membrane, but not on the cytoplasmic side [42]. The two sides of the cell membrane are in different phases, and both sides are at thermodynamic equilibrium [43].

There are significant functional implications from the proposed PLLPI model, compared with the previous models (e.g. the fluid mosaic model). First, outer dense membrane proteins form a robust protein barrier to protect the cell membrane, and these membrane proteins are important to maintain the integrity of the whole cell membrane; otherwise, the lipid layer shown in the fluid mosaic model would be vulnerable to external environmental harm. Second, in the compact protein layer, the conformation of proteins is less flexible than that described in the fluid mosaic model. This accommodates the selectivity and recognition of membrane proteins, such as receptors. Third, the protein domains on the cytoplasmic side of cell membranes work better for energy transfer and signaling transduction. These microdomains are supposed to serve as the platforms for cell signaling and trafficking, which is related to the diversity of lipid and protein composition [14]. Proteins in microdomains may work more efficiently than isolated single proteins described in the fluid mosaic model. Combined with more single molecule approaches, including flourescence recovery after photobleaching (FRAP), fluorescence correlation spectroscopy (FCS), fluorescence resonance energy transfer (FRET) and stimulated emission depletion microscope (STED), researchers may explore more functional merits from the PLLPI model in future studies, regarding the protein/lipid interactions, material transporting and viral infection, etc.

The dense protein layer of the ectoplasmic side of membranes is about 4 nm in height, as measured with AFM. From the currently available X-ray analysis data, the heights of most proteins on the ectoplasmic side of membranes are about 3–10 nm. For instance, the heights of extracellular segments of sodium-potassium pump [44], CD59 [45], carboxypeptidase M [46], G protein-coupled receptor [36], EGFR [47] and T cell receptor [48] are 4.0 nm, 3.2 nm, 6.5 nm, 4 nm, 7.8 nm and 6.1 nm, respectively. Thus, we can assume that these proteins may form a compact layer with a height of 4 nm in the real membrane system. Although we do not know how the proteins are organized on the compact ectoplasmic side of the cell membrane, our result may shed light on protein folding prediction in the real mammalian cell membrane system with the crystal structure of transmembrane proteins.

Some proteins in microdomains of the cytoplasmic side of the cell membrane may be involved in linking the membrane with the cytoskeletal protein, such as actin fiber. Here we show that band 3 proteins could be one of them. Band 3 is verified as the component of the cholesterol-enriched domains from STORM imaging (Figure 5). As reported, band 3 connects with actin filaments via ankyrin to maintain the integrity and stability of the membranes [49]. Therefore, we might speculate that the actin fiber in cells could drive band 3 to control the movement of cholesterol-enriched domains, which is crucial in T cell activation and other membrane functions [50].

In contrast to the fluid mosaic model, the proposed membrane structure(PLLPI)suggests that most proteins in cell membranes diffuse in a more restricted and confined motion. The diffusion of proteins is restricted by many factors, such as protein crowding, cytoskeleton attachment and protein-lipid interactions. Thus membrane liquidity mainly takes place in the intervals between protein domains; in other words, proteins and lipids may only diffuse freely along the borders of the domains. The distances of adjacent protein domains range from 13–105 nm, with the maximum distribution at 53.2±12.0 nm (Figure 4F), which is in agreement with the results reported previously [17].

The membrane structure from other types of cells and organelles, such as primary hepatocytes, crucian carp erythrocytes, human platelets, mitochondrion membrane [51] and Golgi apparatus membrane [52], have also been studied, showing results similar to those of MDCK cells in the present study (Supplemental Information, Figure S4–S8). Meanwhile, we performed conventional Western blotting to detect the differential distribution of membrane integrated proteins, CD47 and Band III (Supporting Information, Figure S9–S11). All these findings demonstrate that our proposed model could be applied to illustrate the basic structure of nucleated mammalian cells, including epithelial or cancer cells, primary or cultured cells, cell membranes or organelle membranes. It is worth noting that this PLLPI model is only suitable for the basic structure of the cell membranes derived from isolated or cultured cells without cellular interactions. It is well known that the cell matrix might also contribute to the complexity of the ectoplasmic side of the cell membrane in living organisms. To address these questions, our future efforts will focus on improving our current model in tissue.

## Materials and Methods

### Cell Culture

We cultured nucleated mammalian cells, including MDCK (Madin-Darby canine kidney), A549 and HeLa cells, on cover slips (Figure 1A). Madin-Darby canine kidney (MDCK) and HeLa cells were purchased from the Shanghai Institute of Biological Sciences. A549 cells (human lung adenocarcinoma cell line) were a generous gift from Prof. Xiaohong Fang (Institute of Chemistry, Chinese Academy of Sciences). MDCK cells were cultured in RPMI 1640 (Hyclone). HeLa and A549 cells were cultured in Dulbecco's modified Eagle's medium (DMEM, Hyclone). All media were supplemented with 10% fetal bovine serum (Gibco), 100 U/mL penicillin, and 100 µg/mL streptomycin. Cells were grown at 37°C in a humidified atmosphere with 5% CO_2_.

### Preparation of Cell Membranes

#### Preparation of the cytoplasmic side of membranes

The cell membranes were prepared as described [53]. Briefly, the cells were washed with 20 mM PIPES and 150 mM KCl (pH 6.2) on ice twice, incubated with ice cold hypotonic buffer (4 mM PIPES, 30 mM KCl, pH 6.2) for 3 min, and then sheared open by a stream of 6 mL of the same buffer through a needle at an angle of 20°. Then they were treated with high- salt buffer (2 M NaCl, 2.7 mM KCl, 1.5 mM KH_2_PO_4_, and 1 mM Na_2_HPO_4_, pH 7.2) for 30 min at room temperature. The prepared membranes were immediately imaged by AFM in PIPES.

#### Preparation of the ectoplasmic side of membranes

Two strategies were used to prepare the ectoplasmic side of membranes. First, cells on the cover slip were sheared open as described above. Second, we incubated the cells in hypotonic buffer and obtained the membranes by centrifugation. Briefly, cells were first incubated with 20 µM cytochalasin B (Sigma) and 60 µM nocodazole (Sigma) for 50 min at 37°C in order to disrupt the actin filaments and microtubules, respectively. Then the cells were digested by 1 mg/mL trypsin and washed with 1 mL PBS (136.9 mM NaCl, 2.7 mM KCl, 1.5 mM KH_2_PO_4_, 8.1 mM Na_2_HPO_4_•7H_2_O, pH 7.4) three times. The cells were treated with 1 mg/mL DNase to disrupt the nuclei/DNAs and then centrifuged at 3000 rpm for 10 min. The supernatant was discarded, and the precipitate was dissolved with PBS and deposited on mica for 1 h for AFM imaging.

### Atomic Force Microscopy

All the AFM experiments were carried out by AFM 5500 (Agilent Technologies, Chandler, AZ) in buffer solutions.

Functionalization of the AFM tips with glutaraldehyde was prepared as described previously [32]. Briefly, the tips were washed with 30% H_2_O_2_/H_2_SO_4_ for h. Then the tips were cleaned with the UV-cleaner in O_3_ atmosphere for 20 min. The tips were then vapor-treated with aminopropyltriethoxysilane (APTES) and immersed in 0.5% (wt) glutaraldehyde PBS solution for 15 min, followed by washing with PBS three times and storage in PBS at 4°C for later use. The force curves were obtained in the force spectroscopy mode in PBS. When the ectoplasmic or cytoplasmic side of membranes was prepared, thousands of force curves were recorded in various positions of different membranes. The force curves were processed with MatLab 7.9 (Math Works Inc.).

All the AFM images were acquired in Acoustic AC (AAC) mode with the bare tips that were conjugated on nonconductive oxide-sharpened Si_3_N_4_ cantilevers with spring constant of 0.01 N/m (nominal) at a scanning rate of 1.5–1.8 Hz. AFM imaging that included the treatment with proteinase K (Sigma), collagenase 3 (Invitrogen) or trypsin was carried out under 37°C by temperature control 325 (Agilent Technologies, Chandler, AZ). Other experiments were performed at room temperature. The images were recorded as 512×512 pixels. The sizes and heights of the membranes and proteins were measured by PicoScan 5.3.3 software (Agilent Technologies, Chandler, AZ).

### Fluorescent Labeling and Fluorescence Microscopy Imaging

#### Membrane and actin filament labeling

After the cytoplasmic side of the cell membranes was prepared as previously described, the samples were fixed with 4% paraformaldehyde, and the actin filaments were labeled with phalloidin-FITC (Beyotime) for 30 min at room temperature. Then the samples were rinsed with PBS three times. The membranes were labeled with 10 µg/mL 1,1'-dioctadecyl-3,3,3',3'-tetramethylindocarbocyanine perchlorate (DiI, Biotium) for 30 min at room temperature, then washed with PBS three times before imaging. All the labeling procedures were conducted in darkness. The fluorescent images were recorded with a Leica SP2 laser scanning confocal microscope. The phalloidin-FITC and DiI were excited with 488 nm Ar-Kr and 543 nm He-Ne lasers, respectively. The emission fluorescent signals were collected and recorded with an NA = 1.40 100× oil-immersion objective.

#### STORM imaging

After the cytoplasmic side of membranes was prepared as described, the membranes were labeled with Cy5-conjugated anti-band 3 antibody. 0.1 µL 10 mg/mL Cy5-n-hydroxysuccinamide (NHS) ester and 5 µL anti-band 3 antibody were diluted in 10 µL 1 M NaHCO_3_ and vortexed for 1 h in darkness. Then the unreacted dyes were filtered out with gel filtration using a mini-spin column (GE Healthcare). The membranes were labeled for 1 h at room temperature. Then the cells were washed with PBS three times. Imaging was carried out in the buffer solution consisting of 50 mM Tris, 10 mM NaCl, 10% glucose (w/v), 0.5 mg/mL glucose oxidase, 40 µg/mL catalase and 1% β-mercaptoethanol (v/v). The prepared cytoplasmic side of membranes were treated with 10 mM MβCD for 30 min to destroy cholesterol-enriched domains and imaged as above. Mannose on the intact unfixed cell surface was labeled with MNA (pure Morniga M lectin, black mulberry, L-9004-1, EY Laboratories, Inc.), and the procedures were similar to those described above. The fluorescent images were recorded with the home-built stochastic optical reconstruction microscopy (STORM) based on Nikon inverted fluorescence microscopy [54]. Cy5 was excited with a 647 nm laser beam, and the emission signal was recorded with an NA = 1.40 100× oil-immersion objective. The images were processed with ImageJ.

## Supporting Information

File S1 **Supporting text and Figures S1–S11. Figure S1.** The distribution of Na^+^-K^+^ ATPase in the inner leaflets of human erythrocyte membranes. Na^+^-K^+^ ATPase was labeled with Na^+^-K^+^ ATPase antibody conjugated with cy5, and the fluorescence image was acquired with STORM. There are a plenty of Na^+^-K^+^ ATPases in the inner leaflet membrane, and the majority of the proteins form microdomains. Scale bar: 2 µm. **Figure S2.** Localizing the EGFR on the outer surface of A549 cells by topography and recognition imaging (TREC). The cell was gently fixed by 4% paraformaldehyde before imaging. EGFR was localized on the surface of A549 cells by scanning the cells with EGF modified AFM tips. (A) The topography of the cell surface shows a relatively smooth feature without protein domain. (B) The corresponding recognition imaging to show the location of EGFRs (dark areas), which indicates that EGFRs exist in the microdomains (about hundreds of nanometers). Scale bar: 500 nm. **Figure S3.** Digestion of the outer leaflet of MDCK cell membranes by PNGase F. The outer leaflet of membranes was treated with PNGase F, which can cleave most of saccharides from glycoproteins. (A) The topography of the outer leaflet membrane treated by PNGase F. There is no pit or indent visible on the smooth membrane. (B) Cross section analysis along the green line in (A), which shows no apparent decrease of the thickness of membranes. Scale bar: 150 nm. **Figure S4.** The outer leaflet membrane of a primary hepatocyte prepared from rat liver. The outer surface is pretty smooth as MDCK cells (Fig.1). Scale bar: 300 nm. **Figure S5.** The outer and inner leaflet membrane of erythrocytes from crucian carp. (A) The outer leaflets of membranes of red blood cell membrane from crucian carp. (B) A whole inner leaflet of red blood cell membrane from crucian carp. There are dense proteins in the inner leaflet membrane. (C) The magnified image from (B). Scale bars: 200 nm in (A), 4 µm in (B), 1 µm in (C). **Figure S6.** The morphology of the outer and inner leaflet of human platelets. (A) The morphology of the outer leaflet of a platelet. (B) The inner leaflet membrane is rough with a plenty of proteins. The proteins are in the status of dispersed domains, which can be clearly observed in the magnified image (C). Scale bars: 100 nm in (A), 1 µm in (B), 500 nm in (C). **Figure S7.** The membranes of mitochondrion from rat liver. (A) The intermembrane space surface of the inner mitochondrial membrane. The membrane surface is very smooth with the roughness of 0.6±0.2 nm. (B) The matrix side of the inner mitochondrial membrane. There are a plenty of proteins in the inner mitochondrial membrane, and they tend to form microdomains. Scale bars: 150 nm in (A), 200 nm in (B). **Figure S8.** The membranes of Golgi apparatus from Hela cells. (A) The smooth outer leaflet membrane of Golgi apparatus. (B) The inner leaflets of membranes are covered with proteins that tend to form dispersed microdomains. Scale bars: 150 nm in (A), 200 nm in (B). **Figure S9.** Western blot analysis of protein differential distribution in Hela cells. (A) Hela cells treated with PBS (ctrl), protease mixture and 0.1% Triton X-100/protease mixture was used as samples. After electrophoresis CD47 monoclonal antibody B6H12 was used as marker for amino acid at the outer membrane leaflets. Compared with control, CD47 band significantly decreased in protease mixture treated sample. Bands of CD47 and actin both disappeared when 0.1% Triton X-100 and protease mixture double treatments were applied. (B) Membrane fraction (mem) or intact Hela cells (total) were used as samples. Band 3 polyclonal antibody targeted to the intracellular N-terminal serves as markers for amino acid at the inner membrane leaflets. The intensity of Band3 was much stronger in the membrane fraction which implied more epitopes were exposed. **Figure S10.** Topology model of CD47 (Brown and Frazier, 2001). **Figure S11.** Topology model of human erythrocyte BandIII (Bonar and Casey, 2008)
(DOC)
