# Supplementary material for: Studying the Nucleated Mammalian Cell Membrane by Single Molecule Approaches
Source: PLoS One. 2014 May 7;9(5):e91595. doi: 10.1371/journal.pone.0091595 (PMC4012985; doi:10.1371/journal.pone.0091595)
Supplement: File S1 — Supporting text and Figures S1–S11. Figure S1. The distribution of Na+-K+ ATPase in the inner leaflets of human erythrocyte membranes. Na+-K+ ATPase was labeled with Na+-K+ ATPase antibody conjugated with cy5, and the fluorescence image was acquired with STORM. There are a plenty of Na+-K+ ATPases in the inner leaflet membrane, and the majority of the proteins form microdomains. Scale bar: 2 µm. Figure S2. Localizing the EGFR on the outer surface of A549 cells by topography and recognition imaging (TREC). The cell was gently fixed by 4% paraformaldehyde before imaging. EGFR was localized on the surface of A549 cells by scanning the cells with EGF modified AFM tips. (A) The topography of the cell surface shows a relatively smooth feature without protein domain. (B) The corresponding recognition imaging to show the location of EGFRs (dark areas), which indicates that EGFRs exist in the microdomains (about hundreds of nanometers). Scale bar: 500 nm. Figure S3. Digestion of the outer leaflet of MDCK cell membranes by PNGase F. The outer leaflet of membranes was treated with PNGase F, which can cleave most of saccharides from glycoproteins. (A) The topography of the outer leaflet membrane treated by PNGase F. There is no pit or indent visible on the smooth membrane. (B) Cross section analysis along the green line in (A), which shows no apparent decrease of the thickness of membranes. Scale bar: 150 nm. Figure S4. The outer leaflet membrane of a primary hepatocyte prepared from rat liver. The outer surface is pretty smooth as MDCK cells (Fig.1). Scale bar: 300 nm. Figure S5. The outer and inner leaflet membrane of erythrocytes from crucian carp. (A) The outer leaflets of membranes of red blood cell membrane from crucian carp. (B) A whole inner leaflet of red blood cell membrane from crucian carp. There are dense proteins in the inner leaflet membrane. (C) The magnified image from (B). Scale bars: 200 nm in (A), 4 µm in (B), 1 µm in (C). Figure S6. The morphology of the ou [file pone.0091595.s001.doc]

Studying the Nucleated Mammalian Cell Membrane by Single Molecule Approaches

Weidong Zhao^1,2,#^, Yongmei Tian^1,2,#^, Mingjun Cai^1,#^, Feng Wang^1^, Jiazhen Wu^1,2^, Jing Gao^1,2^, Shuheng Liu^1^, Junguang Jiang^1^, Shibo Jiang^3,4^,* and Hongda Wang^1,2^,*

^1^ State Key Laboratory of Electroanalytical Chemistry, Changchun Institute of Applied Chemistry, Chinese Academy of Sciences, Changchun, Jilin 130022, China.

^2^ University of Chinese Academy of Sciences, Beijing 100049, China.

^3^ Key Laboratory of Medical Molecular Virology of Ministries of Education and Health, Shanghai Medical College, Fudan University, Shanghai 200032, China.

^4^ Lindsley F. Kimball Research Institute, New York Blood Center, New York, NY 10065, USA.

*Correspondence: [hdwang@ciac.ac.cn](mailto:hdwang@ciac.ac.cn) (H.W.) or [sjiang@nybloodcenter.org](mailto:sjiang@nybloodcenter.org) (S.J.).

^#^ These authors contributed equally to this work.

**Keywords:** cell membrane; nucleated mammalian cells; in-situ AFM; STORM.

We have imaged various membranes by AFM and STORM to support the proposed PLLPI model, including primary hepatocytes, erythrocytes from crucian carp, human platelets, mitochondrion membrane, and Golgi apparatus membrane. Meanwhile, Na^+^-K^+^ ATPases and EGF receptors were localized in the protein domains in the inner and outer leaflets of membranes, respectively. There is no dense carbohydrate layer on the outer leaflet of cell membranes confirmed by PNGase F digestion. Besides these in-situ single molecule techniques (AFM, STORM and SMFS), we further used conventional Western blotting to test the distribution pattern of membrane proteins.

**1. The distribution of Na^+^-K^+^ ATPase in the inner leaflet of human red blood cell membranes by STORM.**


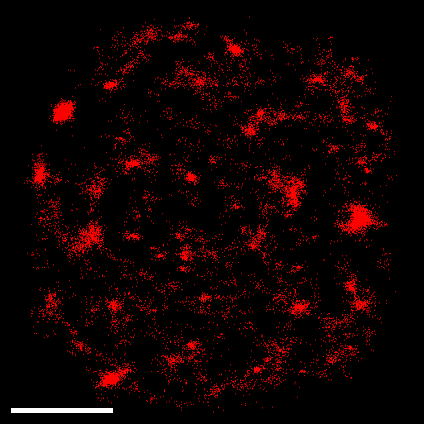


**Figure S1** (Related to Figure 5). The distribution of Na^+^-K^+^ ATPase in the inner leaflets of human erythrocyte membranes. Na^+^-K^+^ ATPase was labeled with Na^+^-K^+^ ATPase antibody conjugated with cy5, and the fluorescence image was acquired with STORM. There are a plenty of Na^+^-K^+^ ATPases in the inner leaflet membrane, and the majority of the proteins form microdomains. Scale bar: 2 μm.

**2. Localizing the EGFR on the outer surface of A549 cells by topography and recognition imaging (TREC).**


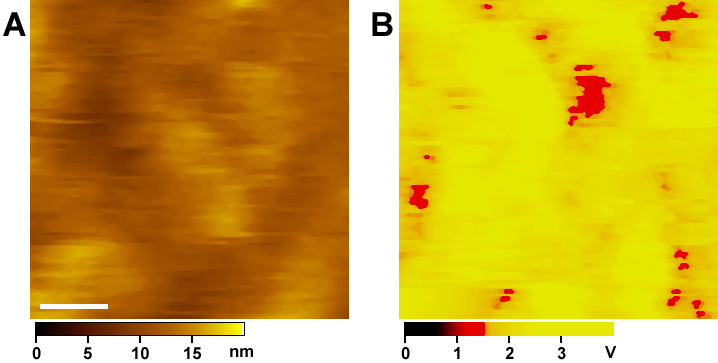


**Figure S2** (Related to Figure 5). Mapping the EGFR on the cell surface by TREC that can recognize target molecules in samples with high precision and specification. The cell was gently fixed by 4% paraformaldehyde before imaging. EGFR was localized on the surface of A549 cells by scanning the cells with EGF modified AFM tips. (A) The topography of the cell surface shows a relatively smooth feature without protein domain. (B) The corresponding recognition imaging to show the location of EGFRs (dark areas), which indicates that EGFRs exist in the microdomains (about hundreds of nanometers). Scale bar: 500 nm.

**3. Digestion of the outer leaflet of MDCK cell membranes by PNGase F**

**
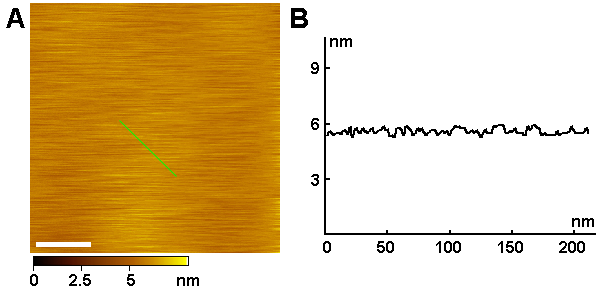
**

**Figure S3** (Related to Discussion). The outer leaflet of membranes was treated with PNGase F, which can cleave most of saccharides from glycoproteins. (A) The topography of the outer leaflet membrane treated by PNGase F. There is no pit or indent visible on the smooth membrane. (B) Cross section analysis along the green line in (A), which shows no apparent decrease of the thickness of membranes.

**4. The outer leaflet membrane of primary hepatocytes from rat.**


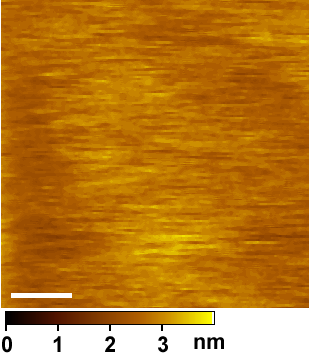


**Figure S4** (Related to discussion). The outer leaflet membrane of a primary hepatocyte prepared from rat liver. The outer surface is pretty smooth as MDCK cells (Fig.1). Scale bar: 300 nm.

**5. The outer and inner leaflet membrane of red blood cells from crucian carp.**


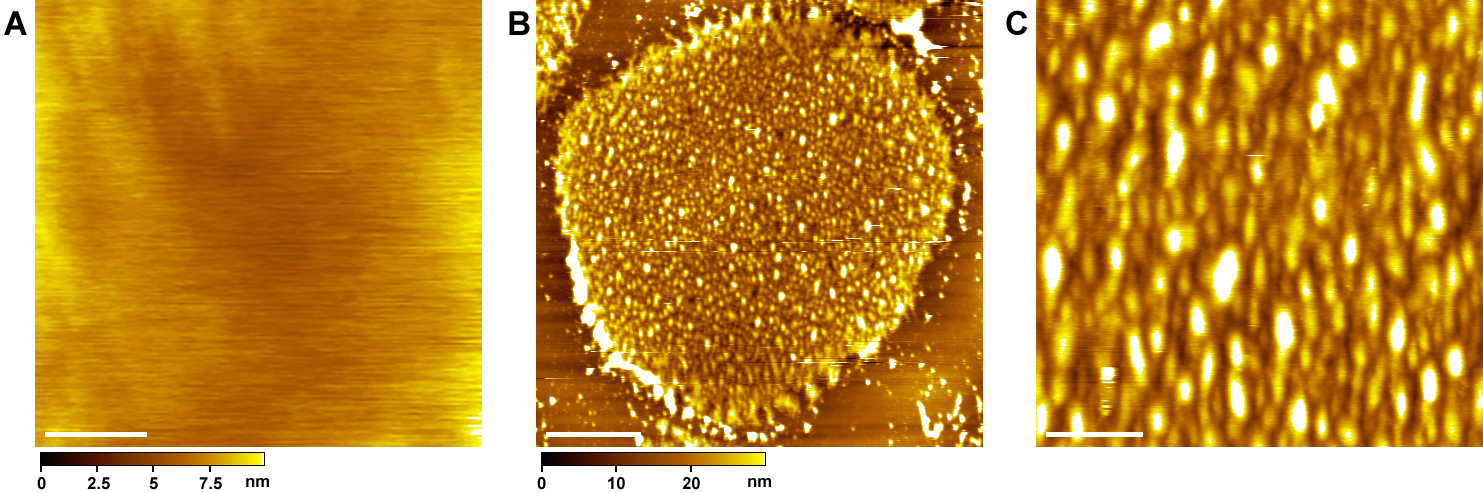


**Figure S5** (Related to discussion). (A) The outer leaflets of membranes of red blood cell membrane from crucian carp. (B) A whole inner leaflet of red blood cell membrane from crucian carp. There are dense proteins in the inner leaflet membrane. (C) The magnified image from (B). Scale bars: 200 nm in (A), 4 μm in (B), 1 μm in (C).

**6. The morphology of the outer and inner leaflet of human platelets.**


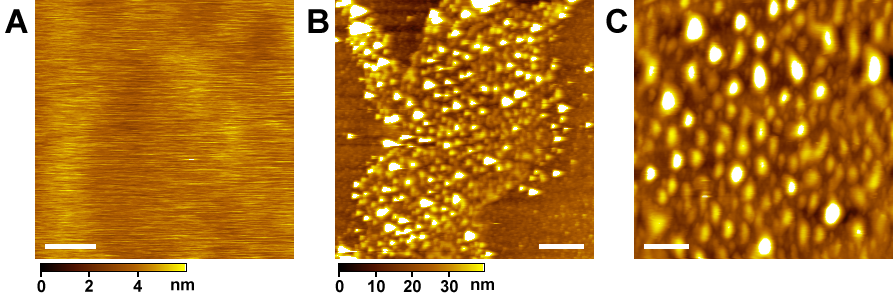


**Figure S6** (Related to discussion). (A) The morphology of the outer leaflet of a platelet. (B) The inner leaflet membrane is rough with a plenty of proteins. The proteins are in the status of dispersed domains, which can be clearly observed in the magnified image (C). Scale bars: 100 nm in (A), 1 μm in (B), 500 nm in (C).

**7. The membranes of mitochondrion.**

.
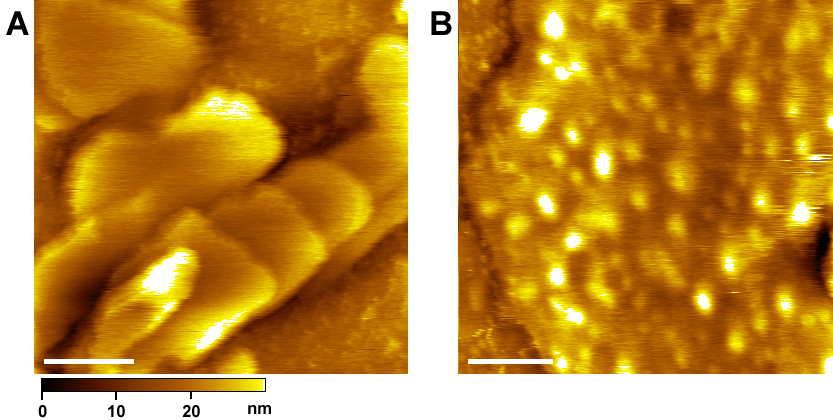


**Figure S7** (Related to discussion). (A) The intermembrane space surface of the inner mitochondrial membrane (from wistar rat). The membrane surface is very smooth with the roughness of 0.6 ± 0.2 nm. (B) The matrix side of the inner mitochondrial membrane. There are a plenty of proteins in the inner mitochondrial membrane, and they tend to form microdomains. Scale bars: 150 nm in (A), 200 nm in (B).

**8. The membranes of Golgi apparatus.**


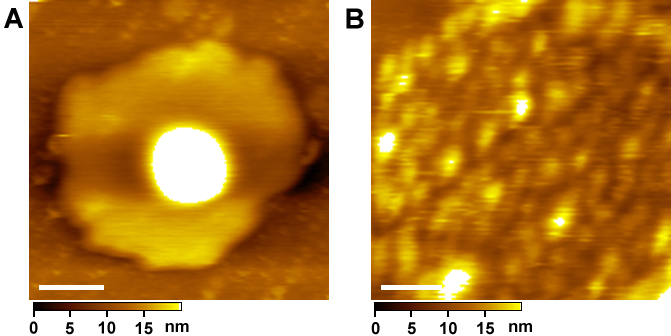


**Figure S8** (Related to discussion). (A) The smooth outer leaflet membrane of Golgi apparatus from Hela cells. (B) The inner leaflets of membranes are covered with proteins that tend to form dispersed microdomains. Scale bar: 150 nm in (A), 200 nm in (B).

**9. Western blot analysis of protein differential distribution in Hela cells.**

Besides the in-situ single molecule techniques (AFM, STORM and SMFS), we also used conventional western blotting to detect the distribution pattern of membrane integrated protein, CD47 and BandIII. CD47 is a type I integral membrane protein composed of an extracellular immunoglobulin variable (IgV)-like domain, five membrane-spanning segments, and an alternatively spliced carboxyterminal cytoplasmic tail [1]. B6H12 (monoclonal antibodies specific for human CD47) targeted to extracellular IgV-like domain, was used as marker for amino acid leaflets at the outer membrane. To determine the extracellular IgV domain of CD47, Hela cells treated with protease mixture (with or without 0.1% Triton X-100) were used as samples. Compared with control sample, CD47 band (approximately 40 kDa) significantly decreased when treated with protease mixture (Fig. S1A). Bands of CD47 and actin both disappeared when 0.1% Triton X-100 and protease mixture double treatments were applied. Band 3 is an anion transporter, both the N and C-terminal domains of which are cytosolic [2]. In our experiment, polyclonal antibody targeted to N-terminal domain was used as marker for amino acid leaflets at the inner membrane. Since the membrane skeleton and intracellular component might hinder the interaction between antibody and corresponding epitope. Membrane fraction of Hela cells was isolated, with same amount of intact cells as control. Our results show that, in comparison with intact cell (total), more epitopes of Band 3 (approximately 100 kDa) were recognized in membrane fraction (mem). In the meantime, the amount of actin was significantly reduced in membrane fraction samples. Our results that the differential distribution pattern of membrane integrated protein at population level, provides the important support for our microscope-based hypothesis.


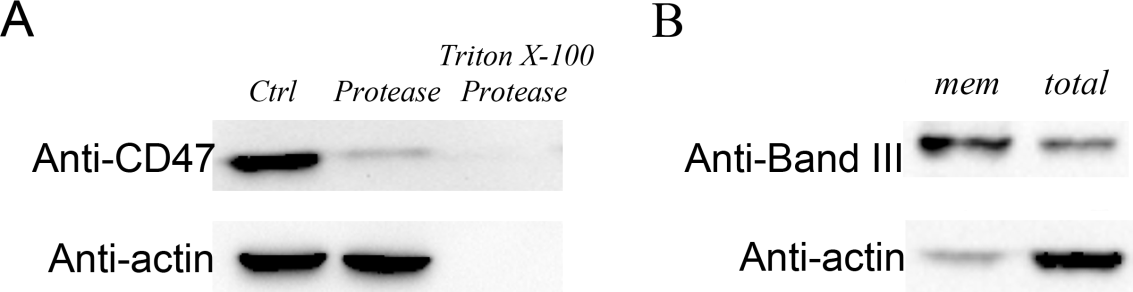
**Figure S9**. Western blot analysis of protein differential distribution in Hela cells. A. Hela cells treated with PBS (ctrl), protease mixture and 0.1% Triton X-100/protease mixture was used as samples. After electrophoresis CD47 monoclonal antibody B6H12 was used as marker for amino acid leaflets at the outer membrane. Compared with control, CD47 band significantly decreased in protease mixture treated sample. Bands of CD47 and actin both disappeared when 0.1% Triton X-100 and protease mixture double treatments were applied. B. Membrane fraction (mem) or intact Hela cells (total) were used as samples. Band 3 polyclonal antibody targeted to the intracellular N-terminal serves as markers for amino acid leaflets at the inner membrane. The intensity of Band3 was much stronger in the membrane fraction which implied more epitopes were exposed.

**Figure S10**. Topology
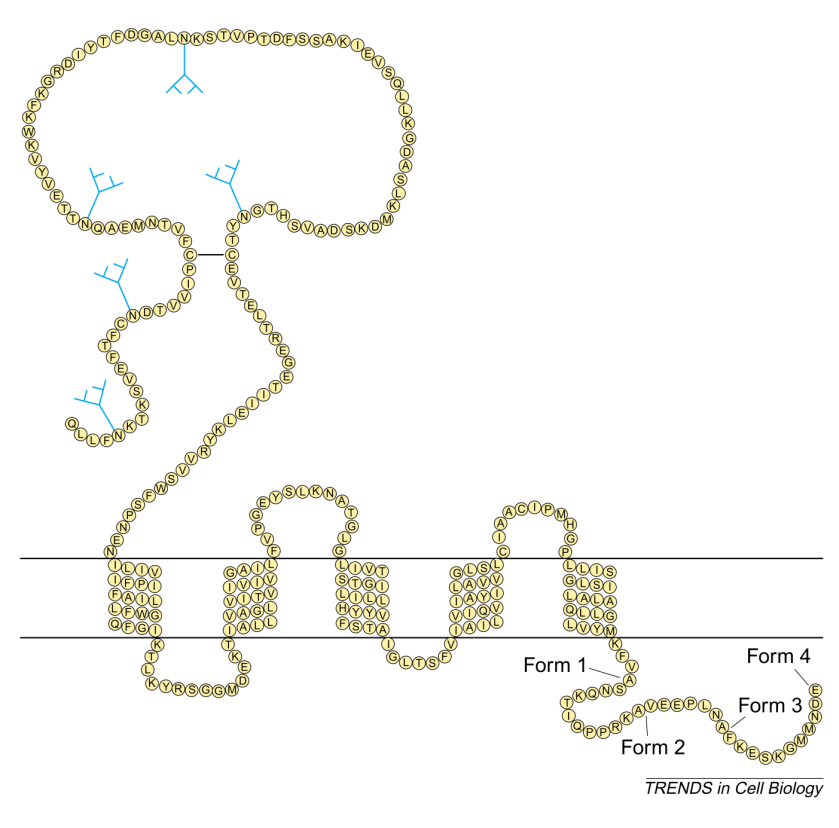
model of CD47 (Brown & Frazier, 2001).


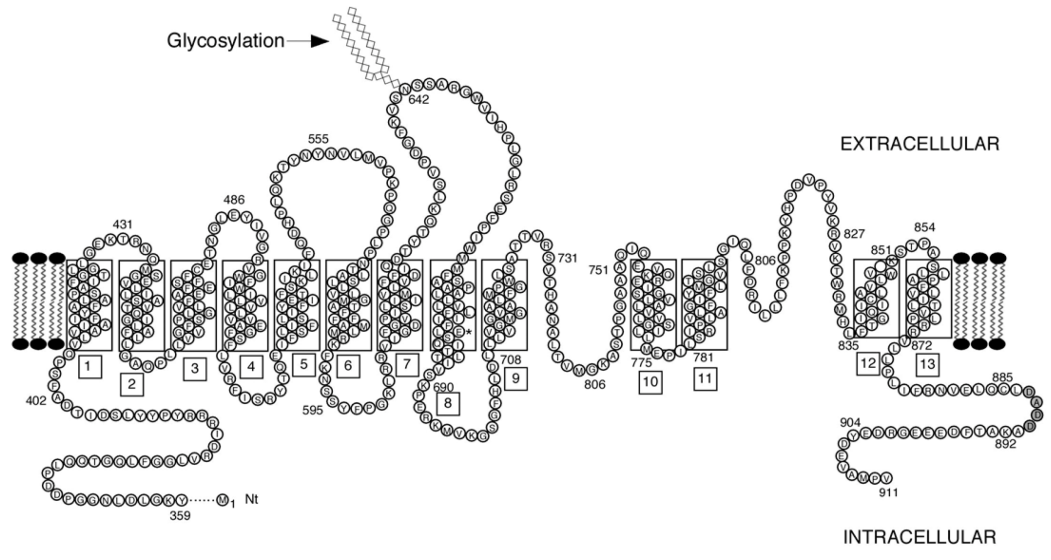
**Figure S11**. Topology model of human erythrocyte BandIII (Bonar and Casey, 2008)

**10. Supplemental materials and methods.**

**Protease Digestion.** Same amount of Hela cells (2×10^6^) were washed with ice-cold phosphate buffered saline (PBS) and incubated for 1 h with either PBS (control) or protease mixture (0.25% trypsin, 20 μg/ml Proteinase K and 5 μg/ml Collagenase) at 37 ℃. Protease digestion was terminated by adding phenylmethylsulfonyl ﬂuoride to a ﬁnal concentration of 1 mM. In some samples, prior to trypsin digestion, Triton X-100 was added to a ﬁnal of concentration of 0.1% to penetrate the membrane and expose the internal structure to the protease. After treatment with protease, Hela cells were washed with PBS for twice.

**Membrane Fraction Isolation.** Incubated the cells in hypotonic buffer and obtained the membranes by centrifugation. Cells were first incubated with 20 μM cytochalasin B (Sigma) and 60 μM nocodazole (Sigma) for 50 min at 37 °C in order to disrupt the actin filaments and microtubules, respectively. Then the cells were digested by 1 mg/mL trypsin and washed with 1 mL PBS (136.9 mM NaCl, 2.7 mM KCl, 1.5 mM KH_2_PO_4_, 8.1 mM Na_2_HPO_4_•7H_2_O, pH 7.4) three times. The cells were treated with 1 mg/mL DNase to disrupt the nuclei/DNAs and then centrifuged at 3000 rpm for 10 min. The supernatant was discarded, and the precipitate was dissolved with PBS for Western blotting.

**Western Blotting.** All samples were dissolved with 300 μl RIPA lysis buffer (Beyotime) and resolved with 8% SDS-PAGE and analyzed by Western blotting. After transferring proteins to Polyvinylidene Fluoride membranes, blots were detected using anti-Band3 poly-clonal antibody (Abcam ab55830, recognized the aminoterminal end, eluted at 1:500) anti-huCD47 monoclonal antibody (BD 561249, B6H12, eluted at 1:500) or anti-actin (Trans, C0014, eluted at 1:1000) and developed using horseradish peroxidase-linked secondary antibodies and the enhanced chemiluminescence detection system (Beyotime, <http://www.beyotime.com/>).

**Reference**

1. Brown, E. J. and W. A. Frazier (2001). "Integrin-associated protein (CD47) and its ligands." Trends Cell Biol 11(3): 130-135.
2. Bonar P. T. and Casey J.R. (2008). "Plasma membrane Cl-/HCO3- exchangers Structure, mechanism and physiology" Channels 2(5): 337-345;
